# Evaluating Personalization: The AB Testing Pitfalls Companies Might Not Be Aware of—A Spotlight on the Automotive Sector Websites

**DOI:** 10.3389/frai.2020.00020

**Published:** 2020-04-09

**Authors:** Maria Esteller-Cucala, Vicenc Fernandez, Diego Villuendas

**Affiliations:** ^1^Universitat Politècnica de Catalunya-Barcelona Tech., Barcelona, Spain; ^2^SEAT, S.A., Barcelona, Spain; ^3^TechTalent-Lab, Department of Management, Universitat Politècnica de Catalunya-Barcelona Tech., Barcelona, Spain

**Keywords:** controlled experiments, online experiments, A/B testing, personalization, online personalization

## Abstract

The importance of companies' website as instrument for relationship marketing activities is well-known both in the academia and in the industry. In the last decades, there has been great interest in studying how technology can be used to influence people's attitudes and motivate behavior change. With this, web personalization has had increasing research and practitioner interest. However, the evaluation of user interaction with companies' websites and personalization effects remains an elusive goal for organizations. Online controlled experiments (A/B tests) are one of the most commonly known and used techniques for this online evaluation. And, while there is clearly value in evaluating personalized features by means of online controlled experiments, there are some pitfalls to bear in mind while testing. In this paper we present five experimentation pitfalls, firstly identified in an automotive company's website and found to be present in other sectors, that are particularly important or likely to appear when evaluating personalization features. In order to obtain the listed pitfalls, different methods have been used, including literature review, direct, and indirect observation within organizations of the automotive sector and a set of interviews to organizations form other sectors. Finally, the list of five resulting pitfalls is presented and some suggestions are made on how to avoid or mitigate each of them.

## 1. Introduction

The importance of companies' website as instrument for relationship marketing activities is well-known both in the academia and in the industry (Mahmoud et al., 2017). In the last decades, there has been great interest in studying how technology can be used to influences people's attitudes and motivate behavior change (Oinas-Kukkonen and Harjumaa, 2008b). Moreover, users are nowadays more likely to look for an emotional connection with the interfaces they come across with (Mendoza and Marasinghe, 2013). Accordingly, companies are not anymore using their websites only to inform about their products or services and sell them, they now need to persuade their users to engage with them (Rashid et al., 2016). With this, the evaluation of user interaction with companies websites is in the spotlight (Spiliopoulou, 2000; Yen et al., 2007).

Regardless of the organization size, website owners try to increase users' interface persuasiveness by adapting colors, texts, or layout (Hohnhold et al., 2015). Following this attempt to be continuously improving, the positive effects of website personalization in company pervasiveness have been gaining attention (Kaptein et al., 2015). Web personalization has been proven not only to have a direct effect on user persuasion (Tam et al., 2005; Oinas-Kukkonen and Harjumaa, 2008a), but also to reduce user reference uncertainty and user obfuscation due to information overload (Arora et al., 2008; Xu et al., 2014; Choi et al., 2017). Moreover, it has been proven to increase trustworthiness perception of the organization, satisfaction, user engagement and user loyalty (indirectly by increasing satisfaction and engagement) (Lee and Lin, 2005; Coelho and Henseler, 2012; Xu et al., 2014; Demangeot and Broderick, 2016; Bleier et al., 2017; Piccoli et al., 2017).

This, among other reasons, has set excellent conditions for web personalization to prosper (Salonen and Karjaluoto, 2016). However, while general personalization effects have been proven by the academia, website personalization is a broad concept and determining the specific impact of particular personalized features on an organization's website remains an elusive goal (Kwon et al., 2010; Kaptein et al., 2015). According to that, recent survey results report that “marketers are more unsatisfied with their current efforts and are less confident in their ability to achieve successful personalization” (Researchscape International and Evergage, Inc.). Therefore, there is still not a consensus on how to measure the persuasive effect of personalization (Kaptein and Parvinen, 2015).

Separately, online controlled experiments (also known as A/B tests) play nowadays a significant role in evaluating the impact that website changes have on users (Das and Ranganath, 2013) being one of the most common methods used (Dmitriev et al., 2016). These two trends, accompanied by its simplicity (Knijnenburg, 2012; Bakshy et al., 2014), have created an increasing use of A/B testing to evaluate personalization features on websites (Amatriain and Basilico, 2012; Dmitriev et al., 2017). Evaluating of personalization improvements has become a popular applications in A/B testing (Fabijan et al., 2016; Govind, 2017; Letham et al., 2018). In the simplest case, the experiment participants of an A/B test are randomly split into either one of two comparable groups. The only difference between the groups is some change or variation *X* deliberately included by the experimenter (from simple changes to personalization algorithms and recommender systems). If the experiment is designed and executed correctly, external factors are distributed evenly between the two groups. Thus, the only thing consistently different between the variants is the change *X*. Hence, any difference in metrics between the two groups must be due to the change *X* or a random change (the second being ruled out using statistical testing). Thereby establishing a causal relationship between *X* and the measured difference in metrics between the two variants (Kohavi et al., 2007; Crook et al., 2009; Fabijan et al., 2016; Zhao and Zhao, 2016; Johari et al., 2017). With the rise of software and internet connectivity, A/B testing presents an unprecedented opportunity to make causal conclusions between the changes made and the customers' reaction on them in near real time (Fabijan et al., 2016). Big players [e.g., Amazon (Dmitriev et al., 2016), Facebook (Bakshy et al., 2014), Google (Hohnhold et al., 2015), Netflix (Amatriain and Basilico, 2012), or Uber (Deb et al., 2018)] as well as smaller companies have been using A/B testing as a scientifically grounded way to evaluate changes and comparing different alternatives (Deng et al., 2016). And, in the last years, the rapid rise of A/B testing has led to the emergence of multiple commercial testing platforms able to handle the implementation of these experiments (Dmitriev et al., 2017; Johari et al., 2017) that, according to the survey results presented in Fabijan et al. (2018b), are used by ~25% of web experimenters.

During the last decade, both scholars and practitioners have been publishing research articles, white papers and blog posts reporting recurrent pitfalls observed in their organizations (Crook et al., 2009; Kohavi et al., 2014; Dahl and Mumford, 2015; Dmitriev et al., 2017). In the specific case of evaluating web personalization and recommender systems, some of these pitfalls become especially recurrent, obscuring the interpretation of results or inducing invalid conclusions. Typically, most of the publications came from big digital companies, such as Microsoft (Dmitriev et al., 2017), Google (Hohnhold et al., 2015), Facebook (Bakshy et al., 2014), Uber (Deb et al., 2018), or Netflix (Amatriain and Basilico, 2012; Su and Yohai, 2019). However, both small-to-medium companies and also big traditional companies are now adopting website experimentation initiatives (Olsson et al., 2017; Fabijan et al., 2018a). From the observation of some of those initiatives in companies of the automotive sector (commonly seen as traditional industrial companies), we identified and reported some critical pitfalls for the reliability of AB tests that were repeated with worrying regularity (Esteller-Cucala et al., 2019).

The objective of this paper is to analyze if the pitfalls identified in the automotive industry are still present across industries. Specifically, we focus on pitfalls that are specially damaging or likely to appear when evaluating personalization features.

The list of pitfalls studied and presented in this paper was firstly obtained from the observation in a company of the automotive sector, and also, the commented pitfalls are limited to the ones considered basic for the implementation of a testing initiative (Kohavi et al., 2009).

In this paper we discuss a list of five experimentation pitfalls. In order to obtain the list, different information sources were used.

## 2. Methods

In order to obtain the list of pitfalls on AB testing we suggest a mixed approach. To this effect, several procedures have been used.

The result of the three first data gathering methods were already shared in a previous work (Esteller-Cucala et al., 2019). In summary, these methods were:

1. General literature review on the topic of AB testing. From this review we obtained the first draft-list of pitfalls.
2. The active participation in a website testing project of a company in the automotive sector let us gather several data from their testing practices. The analyzed company works with multiple websites. At the time of study, more than 10 websites (managed by different teams) were being AB tested. The data collection in this case consisted in test reports and participative observation.
3. In order to examine if the detected pitfalls are specific of the firstly observed company or generalizable across companies of the same sector, the observation was extended to other automotive companies. With this purpose, we collected data from other seven companies in the automotive sector. In this case, the data collection included summaries of their testing projects (in five of the companies), group meetings with six of the companies and open answer surveys in three of the companies.

After those three first steps, we had a list of pitfalls, identified the regular testing practices of real case companies, that was consistent with the literature. In order to study if these pitfalls should be a general concern across sectors, the observation was extended to companies in sectors other than automotive. In this case, the data collection included attending to open presentation of companies explaining their testing initiatives and a set 18 open-ended interviews.

The interviews consisted of, first, three demographic questions in order to know the sector of the company, the position of the respondent, the number of yearly AB tests run and the use of any commercial experimentation tool (the name of the participant as well as the name of the company were kept anonymous). Second, a set of seven questions were made in order to explore the standard experimentation routine in the respondent's company. The specific questions were oriented to inquire about each of the testing pitfalls of the list (without explicitly mentioning the pitfalls). In order to test if the questionnaire was correctly designed and the questions were correctly formulated to detect each of the pitfalls, a pilot respondent was surveyed. The pilot respondent was working in a company with several publications on the topic, with it, the expected answers were known beforehand.

## 3. Results

As previously said, in this paper we are going to present and discuss a set of pitfalls that, even if they need to be kept in mind for any A/B test, they are more likely to appear when trying to assess the effect of personalized features. The pitfalls commented in this section are not only including statistical issues but also testing misconceptions or bad practices.

### 3.1. Evaluation Metrics Selection

According to different reports, marketers expect personalization effect in terms of visitors engagement, customer experience, brand perception and customer loyalty. However, they declare to be measuring the effects of personalization via improvements in conversion rates, click-through rates, revenues and page views, among others (Adobe, 2013; Benlian, 2015; Researchscape International and Evergage, Inc.). The importance of choosing an evaluation metric that really reflects business objectives is not a distinguishing concern of personalization feature experimenters, but one of the general key challenges for organizations that run controlled experiments (Kohavi et al., 2012). Experiments should be evaluated using metrics that reflect business objectives (Dahl and Mumford, 2015), and at the same time be understandable (as simple as possible to interpret the results), interesting to optimize, representative of good website performance (this is not giving positive results when the user experience is worsening) (Crook et al., 2009; Kohavi et al., 2014).

All in all, the evaluation metrics play a key role throughout the experimentation life cycle (design, running, overall evaluation and final decision) (Dmitriev et al., 2017). Therefore, it is recommended for experimenters to keep one single evaluation metric per experiment (Emily Robinson, 2018), agreed upfront (Kohavi et al., 2007), and kept during the whole test (Keser, 2018). Adding secondary objectives to monitor other relevant metrics or to compute complex predictors of long-term results can be a good practice as long as there is a clear unique and fix evaluation metric experiment.

In order to understand the evaluation metrics selection procedures of the different interviewees the question “*How do you choose the evaluation metrics of your tests?*” was directly asked. From both the observation and the interviews results, we can see how, almost every company is using more than one metric for the evaluation of their tests (except from the respondents working for experimentation consulting firms). Most of the respondents report that their companies combine general objectives of the organization and specific goals depending on the test details. With it, declaring a winner version of the test or deciding if the hypothesis is validated can become a difficult task and the final conclusions of the test might be left to the personal interpretation, which is the opposite of what a web testing initiative should stand for Kohavi et al. (2007). Moreover, the results are in line with the Experimentation Growth Model (Fabijan et al., 2018a). The companies with greater experience on AB testing report the use of stable metrics along their experiments, while companies with less experience report sets of evaluation metrics highly dependent on the specific experiment.

The evaluation metric selection might not seem a testing pitfall itself, however, we consider it the cornerstone of an online controlled experiment. If the unique evaluation metric of the experiment is not selected properly, both the utility and the validity of the test can be doubtful.

### 3.2. Determination of the Experiment Length

When using frequentist statistical approaches, the specific length (in time) of the experiment can not be determined in advance, it can only be estimated given a minimum experiment sample size and a predicted average of users (or any other test unit) per time unit. To determine the sample size of the test upfront is one of the most basic premises given for online controlled experiments. However, we have seen how numerous teams continuously monitor their experiments and stop them before the sample size is reached. Accordingly, this is one of the first advices that testing experts give in their papers and online blogs (Kohavi et al., 2007, 2014; Dahl and Mumford, 2015; Dmitriev et al., 2017; Emily Robinson, 2018). Reaching a specific minimum sample size before being able to obtain any result is one of the requirements of the Null Hypothesis Statistical Testing (NHST); nonetheless, this pitfall could turn irrelevant by changing to another statistical interpretation of the results, such as using Bayesian Hypothesis Testing or Sequential Hypothesis Testing (Deng et al., 2016; Johari et al., 2017; Su and Yohai, 2019) which have been attracting research interest as alternatives to NHST and are already used by some commercial testing tools [e.g., VWO and AB Tasty are based on Bayesian calculations (Stucchio, 2015; Wassner and Brebion, 2018) and Optimizely uses Sequential hypothesis testing (Rusonis and Ren, 2018)]. However, both the performed observations, interviews and previous authors report that frequentist approaches are still the most commonly used for A/B testing (e.g., Kohavi et al., 2007; Emily Robinson, 2018).

It is important to note that this pitfall is not only related to the early stopping of the experiment but also with the post-test segmentation. In order for the results to be valid, the minimum sample size required for the analysis is calculated. This is, if the minimum calculated sample size is *X*, the sample size of any post-test segmentation which is smaller than *X* is not be valid (Keser, 2018).

On the other side using much longer samples than needed can also arise in some experiment difficulties (Dmitriev et al., 2016). Sometimes, constrains to the experiment length are set in order to dissipate temporal effects, such as hour-of-day effects (Su and Yohai, 2019), day-of-week effects (Kohavi et al., 2007), business cycles (QuickSprout, 2019), or seasonality effects (Dmitriev et al., 2016). However, these effects might not impact all the organization or all the tests (Su and Yohai, 2019). In the specific case of a personalized feature depending on temporal factors (e.g., hour of the day) experimenters should consider whether to make a generalization or a case dependent experiment.

For the observed cases of the automotive sector, this was a relevant pitfall because there is only one of the observed companies using a non-frequentist approach. However, no-companies where calculating the sample size beforehand. Regarding the interviewees from companies form other sectors, there is a mix of companies calculating and not calculating the sample size required for the test in advance.

### 3.3. Multiple Comparison Problem

Even if the simplest case of A/B testing is considered when comparing only two variants (one against the control), there is no limit of variants to be compared in a single A/B test (also known as A/B/n test). For example, a common case in personalization is to test complex differences between variants, for this, one recommended approach is to test a collection of different variants including small or independent changes in order to be more precise in determining the specific effects of each variation included (Kohavi et al., 2014). This might also be the case when trying to adjust the personalization algorithms' parameters (Letham et al., 2018) or the individualization degree of the personalization (Arora et al., 2008). In this cases, testing a set of different variants is a good practice, even thought there is a statistical consideration to keep in mind when including more than two variants in an A/B test. When the sample size is calculated for a given significance level (e.g., 10%, equivalent to a 90% confidence level) each comparison has a false positive rate equal to the significance level. If we make multiple comparisons within the same test, the whole-test false positive rate is higher. For example, when trying to compare among 15 variants, the chance of getting a false positive (51%) is almost equivalent to flipping a coin and getting a head (Esteller-Cucala et al., 2019). Moreover, this effect should be taken into account any time that there is more than one comparison in the test (e.g., if more than one metrics monitored within the test or if the test is studied separately for different user segments). Nevertheless, some adjustments have been proposed in the literature (e.g., Bonferroni correction) in order to avoid this pitfall (Kohavi et al., 2007; Dahl and Mumford, 2015; Emily Robinson, 2018).

In order to see if the multiple comparison problem was an experimentation pitfall generally affecting to companies both in the automotive sector and in other sectors, the interview directly included a question asking if experiments with more than two variant were performed within the interviewee's company and, in if this was the case, respondents were asked if any criteria was used in order to adapt the experiment length. The results show that even if not all companies are familiar with more-than-two-variants experiments (specially the observed companies of the automotive sector), it is an extended practice and two thirds of the participants are testing with more than two variants. However, only a minority of respondents were aware of any existent corrections to be applied when conducting multiple comparison tests (apart from the consultancy companies).

### 3.4. Balance Among Experiment Samples

As above mentioned, the main objective with an A/B test is to establish a causal relationship between the test condition and a measurable change in some evaluation metric. This causal relationship is based on the premise that any external alteration to the metrics (except the tested ones) are controlled by the randomization and balanced between the test variants (Zhao and Zhao, 2016). Even if this balance condition is necessary for the test to be reliable, there is still lots of practitioners dismissing its importance. The unbalanced sampling refers to the situation where the split of users between variants does not satisfy the expected ratio (Dmitriev et al., 2017; Emily Robinson, 2018). In extremely unbalanced tests problems, such as the Simpson's paradox might appear (Crook et al., 2009). Due to this, unbalanced sampling is one of the most commented pitfalls (Crook et al., 2009; Dmitriev et al., 2017; Emily Robinson, 2018).

Some unbalance common causes are, for example, changing the sample ratio during the experiment (e.g., using ramp-ups to activate the test), post-test segmentation, post-test grouping of samples tested with different ratios or bugs in the implementation (e.g., a bug that affects only to a specific browser) (Kohavi et al., 2007; Crook et al., 2009; Keser, 2018).

Even though unbalanced sampling is a common pitfall in A/B testing, in websites where personalization is used it gets even more common (both when testing personalized or non-personalized features) (Das and Ranganath, 2013). For the specific case of personalization using *monitoring segments* is recommended. This is, to use segments that are not going to be used for making decisions about the result itself but to ensure that all the relevant distinguishable groups included in the personalization algorithms are distributed between variants according to the test ratio (e.g., segments based in scoring intervals). This technique is known as the stratified sampling (Urban et al., 2016; Keser, 2018).

As seen, there are different causes for unbalanced sampling. In order to not induce specific answers from the interviewees the questions regarding this pitfall were focused on two of the possible causes. First, we asked to the participants if they were using ramp-ups or other secure actuation methods in their tests. The result show that almost no respondents are using these methods, so we can conclude that they are not unbalancing the sampling this way. Second, we asked to the participants if they were regularly using AA tests in order to validate their testing tools [recommended practice to detect bugs that cause unbalance (Zhao and Zhao, 2016)]. The result show that <50% of the respondents report using these kind of validation tests in a regular basis. Even if we know that this pitfall appears in the automotive sector and is consistent with the literature, with the previous two questions we can not extract a conclusion about the generalizability of this pitfall.

### 3.5. Blind Adoption of Good Results

Even if A/B testing is one of the simplest evaluation techniques used for the evaluation of website performance (Knijnenburg, 2012; Bakshy et al., 2014), there are many variables that can affect the results. When the result of a test is unexpectedly bad (e.g., the new feature being tested under-performs by long the previous one) a frequent response is to look for the bug. On the contrary, this behavior is not as common when the unsuspected result is good. In the literature, this is known as “failing to apply Twyman's Law” (Dmitriev et al., 2017). A similar case is when a borderline *p*-value is given as a result from a test (Kohavi et al., 2014). But also, there is a common practice of activating new variants after a non-significant test result because “it doesn't hurt” (Emily Robinson, 2018). Even if each organizations might have different results, authors claim that only one-third of the experiments performed in their company improved the metrics they were designed to improve (Kohavi et al., 2014).

When thinking on the scientific rigor assumed for web experimentation, one may presume that this specific pitfall might be unlikely to happen in real organizations. However, as reported in Esteller-Cucala et al. (2019), we had the chance to see several times how some tests are prepared with high expectations of obtaining a specific result. After the collection of interviews we have seen that approximately half of the interviewees companies directly apply the new variant in case of a winning result. Seen the number of pitfalls that are not commonly considered when A/B testing, directly applying the results without more test iterations might result in the activation of false winners (false positive results), borderline *p*-values, insufficient sample sizes tested and so on. Some other respondents report an analysis of secondary metrics to decide whether to activate the winner variant or not.

In the specific case of testing personalization, it might be more difficult to deduct whether a given result makes sense or not, making it easier for some incorrect results to go unnoticed. For these reasons, even if the blind adoption is general a pitfall in A/B testing, it is even more likely to appear in the specific case of testing personalized features. Considering the double-check (or even *double-test*) of the test results might, in some cases, not only be a good practice but also a requirement especially in tests reporting unexpected or borderline results.

## 4. Discussion and Conclusions

While there is value in evaluating personalized features by means of online controlled experiments (A/B tests), there are some pitfalls to bear in mind while testing websites. In this paper, we discuss some critical AB testing pitfalls that were firstly identified in automotive companies and may compromise the validity of their experiments. Moreover, the analysis is then extended to study the presence of this testing pitfalls in companies from sectors other than automotive (always keeping the focus of the study on small-to-medium companies and also big traditional companies with relatively recent adoption of web testing initiatives). As a result, we presented five pitfall topics and commented their presence in the different sectors studied.

After a decade of publications from expert practitioners and big digital companies, the most basic and critical pitfalls are substantially well-documented. Despite this, companies adopting AB testing seem not being completely aware of this testing pitfalls. As seen in the results of this study, most of the respondent companies have not a clear procedure for the selection of their evaluation metrics, which is the starting point of an AB test. Moreover, a remarkable number of the surveyed companies directly apply winner results without further analysis of the test (blind adoption of good results) and are not aware of the multiple comparison problem and its possible corrections to take it into account. However, even if there is still a noteworthy proportion of companies not determining the experiment length beforehand (when using frequentist statistical approaches) the results for the general industries surveys are better than in the companies of the automotive sector. Finally, regarding the pitfalls with the balance among experiment samples, the answers gotten from the survey are not clear enough to extract a conclusion. With it, our results show that there are some basic AB testing pitfalls, well-known by scholars and big digital companies, that are present in the experimentation initiatives of companies relatively inexpert with AB testing.

As previously stated, the list of pitfalls included in this study is by no means the complete list of possible pitfalls that may appear when performing AB tests or even the complete list of pitfalls that can be collected by reviewing the literature. Other pitfalls are still commonly seen in companies and may appear while running specific tests. Some examples are: not considering temporal effects on the user behavior (e.g., holiday seasons or Valentine's Day), neglecting novelty effects or ignoring temporal cycles (both business or calendar cycles) (Kohavi et al., 2007; Dmitriev et al., 2016, 2017; Weinstein, 2019). Even though those pitfalls are also important and should be studied in order to verify the reliability of each result, they might not apply for each test and company (Su and Yohai, 2019). The pitfalls commented in this paper are limited to the ones observed in a specific company of the automotive sector (and then validated with other companies) and the ones considered critical for the validity of the test. If they are not understood and addressed properly, these pitfalls might invalidate not only specific test but the entire testing initiative of a company.

However, this study has some limitations, here we point out four of them. First, the list of testing pitfalls commented in this paper was firstly focused on the automotive industry, therefore, some important pitfalls for other sectors might be missing. Second, the list here presented is not a complete list of possible web AB testing pitfalls, but a list of the observed ones that are considered the most basic and critical for the global testing initiative of a company. Third, the total number of analyzed companies is not large enough to statistically determine the generalizability of each of the presented testing pitfalls. Further research could extend the study to a larger group of companies. Finally, this work is only focused on examining the presence of these testing pitfalls across the industry. However, the reasons why these testing pitfalls can still be found inside the companies, despite the large body of knowledge available on how to identify and avoid them, are not studied and could be addressed on further research.

Additionally, further work needs to be done in the experimentation procedures organizations use to evaluate their personalization efforts. With it, we propose to organizations to construct their own evaluation framework. This is, inspired in the most common pitfalls reported in A/B testing, organization could set the conditions for their teams to experiment. This framework should include for example, the criteria for the evaluation metrics selection, the criteria to be used in order to determine the experiments length (not determining the specific length but setting the criteria to determine it), post-test segmentation criteria and results adoption criteria.

With this work, we aim to increase the experimenters' awareness on those pitfalls. And also, to attract the attention of persuasive technology scholars on the gap between academia advances on the personalization field and its adoption on the industry.

## Data Availability Statement

The datasets generated for this study will not be made publicly available. The interview data might contain personal data that has been omitted in the article.

## Ethics Statement

Ethical review and approval was not required for the study on human participants in accordance with the local legislation and institutional requirements. Written informed consent from the participants was not required to participate in this study in accordance with the national legislation and the institutional requirements.

## Author Contributions

ME-C, VF, and DV contributed to the design. ME-C implemented the research, the analysis of the results and the writing of the first manuscript. VF and DV supervised the project. ME-C, VF, and DV contributed to the final version of the manuscript.

### Conflict of Interest

ME-C and DV were employed by the company SEAT, S.A. The remaining author declares that the research was conducted in the absence of any commercial or financial relationships that could be construed as a potential conflict of interest.
